# Steps towards more integrated care in New Zealand: a general practice perspective

**DOI:** 10.3399/bjgpopen17X100845

**Published:** 2017-03-15

**Authors:** Les Toop

**Affiliations:** Head of Department of General Practice, University of Otago, Christchurch, New Zealand

**Keywords:** general practice, integration, alliancing, New Zealand

The imperative to better integrate health care in New Zealand started a quarter of a century ago and has accelerated in recent years, some of it recently showcased at the 4th World Congress on Integrated Care in 2016.^1^ There exist mature models of horizontal integration, patchy examples of effective vertical integration, and much talk from the highest levels of government of the need for more intersectoral integration. The concept of improving horizontal integration by co-location and collaboration is of course far from new, with many and varied primary care workers in New Zealand expressing the wish to develop closer working relationships in the 1990s.^2^ These collaborative sentiments were not always shared by national disciplinary leaders and politicians.^3^ The integrated family health centre or healthcare home model with innovative models of care is only now gaining momentum in parts of New Zealand^4^, but is yet to be trialled and evaluated at scale.

## First steps for organised general practice in New Zealand

In the 1990s, competition in health was enshrined in policy by a hard-line, right-wing government with public hospitals rebranded as 'Crown Health Enterprises'. They were instructed to compete against each other and to deliver profits.^5^ Meanwhile, general practice was organising itself into collaborative independent practice associations (IPAs) to share after hours care, to negotiate collectively, to take back control of their own continuing education, to provide support for evolving computerisation, and to work together on other areas of mutual interest. The opportunity for several nascent IPAs to take on risk-sharing budgets for selected referred services, (principally medicines and laboratory tests), arose from a policy vacuum on primary care direction, spiralling expenditure and innovative thinking from the sector. In some places, budget holding proved to be very successful in the early years and provided those IPAs with the financial resources to begin developing more integrated services. Importantly, these initiatives were designed, owned, and implemented from the ground up. In Canterbury, the Pegasus IPA was formed in 1992. It was, and remains, the largest organised general practice network in the region. Pegasus quickly developed and self-funded a range of initiatives, including peer-led interdisciplinary education and quality improvement, together with a number of population health programmes, most of which continue today.^6^ Vertical integration was given a further boost with the introduction of an acute demand programme in 2000, which delivered care for selected higher acuity patients in the community, who previously would have been hospitalised. The key success factor was allowing general practice teams to decide what resources and services were required to safely replace a hospitalisation with care in the community. Coordination was provided by Pegasus and an immediate, no-questions-asked approach to funding approval achieved enthusiastic uptake and rapid results in decreased acute medical hospitalisations. This highly successful community-governed model, contrasting with the hospital-in-the-home outreach model much used in Australia, is now in its 17^th^ year. It has established many local community partnerships, works closely with secondary care, and has been replicated extensively around New Zealand.^7^

In 2001, the now centre-left government launched a *Primary Health Care Strategy* ^8^ that, for the first time, encouraged universal patient enrolment with part payment of fees under a capitated formula. Most funding was to be distributed through new entities named primary health organisations (PHOs), which were allowed to form themselves from willing participants, but which were required to have community involvement in governance. Beyond the obvious advantages of the mutual responsibility that comes with enrolment, this change encouraged closer and more formal working relationships between primary care clinicians, community agencies, and non-governmental organisations.

## Introducing the concept of alliancing: shared problems, shared solutions

Moving on a few years, and a change back to a centre-right government, it was becoming clear that the pace of change needed to increase to meet the changing demographics (of both the population and the health workforce) to address unacceptable inequities in health outcomes between the most and least advantaged. In response to a new policy document introduced in 2009, called *Better, Sooner, More Convenient* ^9^ volunteer groups, mostly in the geographic distribution of district health boards, submitted plans to develop more integrated services. The concept of alliancing was encouraged, and in Canterbury, this approach was adopted enthusiastically with the formation of the Canterbury Clinical Network. The measurable benefits of this new way of working and planning have been substantial, and some of these, together with their detailed workplan are publicly available.^10^ Acceleration and incentive was provided by the destructive Christchurch earthquakes in 2010–2011, which significantly reduced hospital inpatient capacity. The key success to the alliancing approach is seen as the commitment from all partners to own the problems, and plan and work together to find solutions. Clinicians from across the system work with funders, and multiple community agencies to redesign services with a shared focus on organising care that is integrated to provide 'best-for-patient', 'best-for-system' outcomes. Nationally, productive alliancing activity has been patchy, but there are some notable successes in progressing integrated care detailed on the General Practice NZ website.^11^ In parallel, horizontal and vertical integration have been facilitated by a number of collaborative IT developments, between general practice, secondary care, and local funders. HealthPathways^12^ and HealthInfo^13^ are prime examples. Developing the content of the care and referral pathways fosters strong primary and secondary care relationships. Locally adapted healthpathways have now been extended to most of New Zealand, much of Australia, and now has a foothold in the North East of England. HealthOne^14^ is a shared electronic record view of many parts of the health system (primary care, secondary care — inpatient and outpatient —﻿ pharmacy, radiology, laboratory, community nursing, and others). This platform will soon extend the visibility or relevant parts of clinical records to the whole South Island of New Zealand.

## The beginnings of intersectoral integration

The current New Zealand government, championed by the new prime minister, is very enthusiastic about developing greater intersectoral collaboration, and has identified the need for, and commitment to, social investment in the most vulnerable and at risk populations who repeatedly require the services and attention of social welfare, education, and justice systems.^15^ Early social sector trials, which allow increased local autonomy in social service provision, are being evaluated.^16^ The recent introduction of a co-developed national system level measures framework to replace the previous single disease-based pay-for-performance programme, should also add incentive to integration organised through local alliances.^17^

In Canterbury, both horizontal and vertical integration are developing.^18^ Comprehensive intersectoral integration seems inherently sensible. Whether it is practically achievable, or simply a chimera, remains to be seen.
